# Ultrasound-Assisted Synthesis of Luminescent Micro- and Nanocrystalline Eu-Based MOFs as Luminescent Probes for Heavy Metal Ions

**DOI:** 10.3390/nano11092448

**Published:** 2021-09-20

**Authors:** Stefaniia S. Kolesnik, Viktor G. Nosov, Ilya E. Kolesnikov, Evgenia M. Khairullina, Ilya I. Tumkin, Aleksandra A. Vidyakina, Alevtina A. Sysoeva, Mikhail N. Ryazantsev, Maxim S. Panov, Vasiliy D. Khripun, Nikita A. Bogachev, Mikhail Yu. Skripkin, Andrey S. Mereshchenko

**Affiliations:** 1 Saint-Petersburg State University, 7/9 Universitetskaya emb., 199034 St. Petersburg, Russia; staphylinuscaesareus@gmail.com (S.S.K.); nosoff.vitia2018@yandex.ru (V.G.N.); ilya.kolesnikov@spbu.ru (I.E.K.); iskint@mail.ru (E.M.K.); i.i.tumkin@spbu.ru (I.I.T.); vidyakina.aleksandra@mail.ru (A.A.V.); mikhail.n.ryazantsev@gmail.com (M.N.R.); m.s.panov@spbu.ru (M.S.P.); v.khripun@spbu.ru (V.D.K.); allanfrack@yandex.ru (N.A.B.); skripkin1965@yandex.ru (M.Y.S.); 2 Sirius University of Science and Technology, 1 Olympic Ave, 354340 Sochi, Russia; sysoevaaa_04@mail.ru; 3 Laboratory of Nanobiotechnology, Saint Petersburg Academic University, ul. Khlopina 8/3, 194021 St. Petersburg, Russia

**Keywords:** metal-organic framework, luminescence, rare earth, europium, nanoparticle, luminescent probe

## Abstract

The luminescent coarse-, micro- and nanocrystalline europium(III) terephthalate tetrahydrate (Eu_2_bdc_3_·4H_2_O) metal-organic frameworks were synthesized by the ultrasound-assisted wet-chemical method. Electron micrographs show that the europium(III) terephthalate microparticles are 7 μm long leaf-like plates. According to the dynamic light scattering technique, the average size of the Eu_2_bdc_3_·4H_2_O nanoparticles is equal to about 8 ± 2 nm. Thereby, the reported Eu_2_bdc_3_·4H_2_O nanoparticles are the smallest nanosized rare-earth-based MOF crystals, to the best of our knowledge. The synthesized materials demonstrate red emission due to the ^5^D_0_–^7^F_J_ transitions of Eu^3+^ upon 250 nm excitation into ^1^ππ* state of the terephthalate ion. Size reduction results in broadened emission bands, an increase in the non-radiative rate constants and a decrease in both the quantum efficiency of the ^5^D_0_ level and Eu^3+^ and the luminescence quantum yields. Cu^2+^, Cr^3+^, and Fe^3+^ ions efficiently and selectively quench the luminescence of nanocrystalline europium(III) terephthalate, which makes it a prospective material for luminescent probes to monitor these ions in waste and drinking water.

## 1. Introduction

Rare-earth-based metal-organic frameworks (MOFs) are actively used in various fields of science and technology as luminescent sensors [1,2,3,4,5,6,7,8,9,10,11], LED components [12], luminescent probes for bioimaging [13,14], and luminescent thermometers [15,16]. Small-sized crystals of the rare-earth-based MOFs are especially interesting due to their unique properties. Such materials have a large specific surface area, and as a result, they can effectively adsorb other ions and molecules, which is necessary for the development of sensitive luminescent sensors [17,18,19]. The presence of heavy metals in drinking water can cause numerous disorders and diseases of humans and animals [20,21]. Therefore, one must develop new sensors for such pollutants. MOFs are actively used as luminescent and electrochemical sensors for heavy metal ion detection in drinking and wastewater [1,2,3,6,7,10]. Nanosized luminescent MOFs are able to penetrate the cell membrane and are therefore used in bioimaging as luminescent probes [13,14]. The nano-sized rare-earth-based MOFs can be synthesized by several synthetic routes [13,14,22,23,24,25] such as solvothermal, reverse microemulsion, surfactant-assisted, microwave, and ultrasonic methods. The resulting small-sized particles usually have sizes from 40 to 5000 nm.

In our current study, we report the room-temperature ultrasonic-assisted wet chemical method of the synthesis of the small-sized luminescent Eu_2_bdc_3_·4H_2_O MOFs including 8 nm nanoparticles—the smallest nanosized rare-earth-based MOF crystals, to the best of our knowledge. The luminescent properties of the coarse-, micro- and nanocrystalline europium(III) terephthalate are studied. In addition, the selective luminescence quenching by heavy metal ions is also reported.

## 2. Materials and Methods

### 2.1. Reagents

Europium chloride hexahydrate was purchased from Chemcraft (Russia). Benzene-1,4-dicarboxylic (terephthalic, H_2_bdc) acid (>98%) sodium hydroxide (>99%), polyethylene glycol 6000 (PEG-6000, for synthesis), iron(III) chloride hexahydrate (>99%), iron(II) sulphate heptahydrate (>99%), chromium(III) chloride hexahydrate (>99%), magnesium chloride hexahydrate (>99%), nickel(II) chloride hexahydrate (>99%), lead(II) nitrate (>99%), cobalt(II) chloride hexahydrate (>99%), anhydrous zinc chloride (>98%), cadmium chloride hydrate (>98%), barium chloride dihydrate (>99%), copper(II) chloride dihydrate (>99%), and EDTA disodium salt (0.1M aqueous solution) were purchased from Sigma-Aldrich Pty Ltd. (Germany) and used without additional purification. The 0.2 M solutions of the above-mentioned salts were prepared and standardized by complexometric titration with EDTA. An amount of 0.3 moles of the terephthalic acid and 0.6 moles of the sodium hydroxide were dissolved in the distilled water to obtain 1 L of 0.3 M solution of the disodium terephthalate (Na_2_bdc).

### 2.2. Synthesis

The europium(III) terephthalate was obtained by mixing the EuCl_3_ and Na_2_bdc solutions. Sample **1** was synthesized by a slow mixing of equal volumes of the 2 mM Na_2_bdc and 1 mM EuCl_3_ solutions accompanied by vigorous stirring (Table 1). Sample **2** was synthesized by a slow mixing of the equal volumes of the 2 mM Na_2_bdc solution and the solution containing 1 mM EuCl_3_ and 20% PEG-6000, accompanied by ultrasonication (40 kHz, 60 W) and vigorous stirring. The white precipitates of europium(III) terephthalate (Samples **1** and **2**) were separated from the reaction mixture by centrifugation (4000× *g*) and washed with deionized water 5 times. Sample **3** was synthesized by a slow mixing of equal volumes of 1 mM Na_2_bdc and 0.5 mM EuCl_3_ accompanied by ultrasonication (40 kHz, 60 W) and vigorous stirring. The obtained clear solution was centrifugated at 7500× *g*; however, no solid was precipitated. The addition of both polar (methanol and acetone) and non-polar solvents (ethanol–dichloromethane mixture) did not result in the salting-out of any solid. Therefore, we used the solution of Sample **3** in the further experiments. All experiments were performed at the temperature of 25 °C.

### 2.3. Characterization

The morphologies of the microstructures of the synthesized Samples **1** and **2** were characterized using scanning electron microscopy (SEM) with a Zeiss Merlin electron microscope (Zeiss, Germany) equipped with the energy-dispersive X-ray spectroscopy (EDX) module (Oxford Instruments INCAx-act, UK). X-ray powder diffraction (XRD) measurements were performed on a D2 Phaser (Bruker, USA) X-ray diffractometer using Cu Kα radiation (λ = 1.54056 Å). The particle size distribution of the aqueous solution of Sample **3** was revealed by the dynamic light scattering technique with an SZ-100 Series Nanoparticle Analyzer (Horiba Jobin Yvon, Japan) The luminescence spectra were recorded with a Fluorolog-3 fluorescence spectrometer (Horiba Jobin Yvon, Japan). Lifetime measurements were performed with the same spectrometer using a pulsed Xe lamp (pulse duration 3 µs). The absolute values of the photoluminescence quantum yields were recorded using a Fluorolog 3 Quanta-phi device. All measurements were performed at the temperature of 25 °C.

## 3. Results and Discussion

### 3.1. Morphology

**Figure 1:**
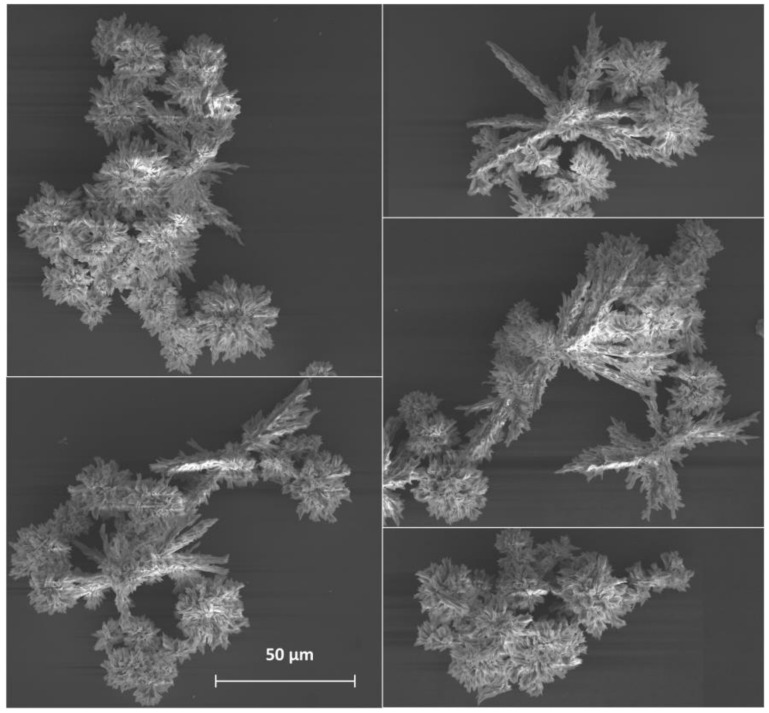
SEM images of Sample **1**. The average diameter of polycrystals is 120 ± 30 µm.

**Figure 2:**
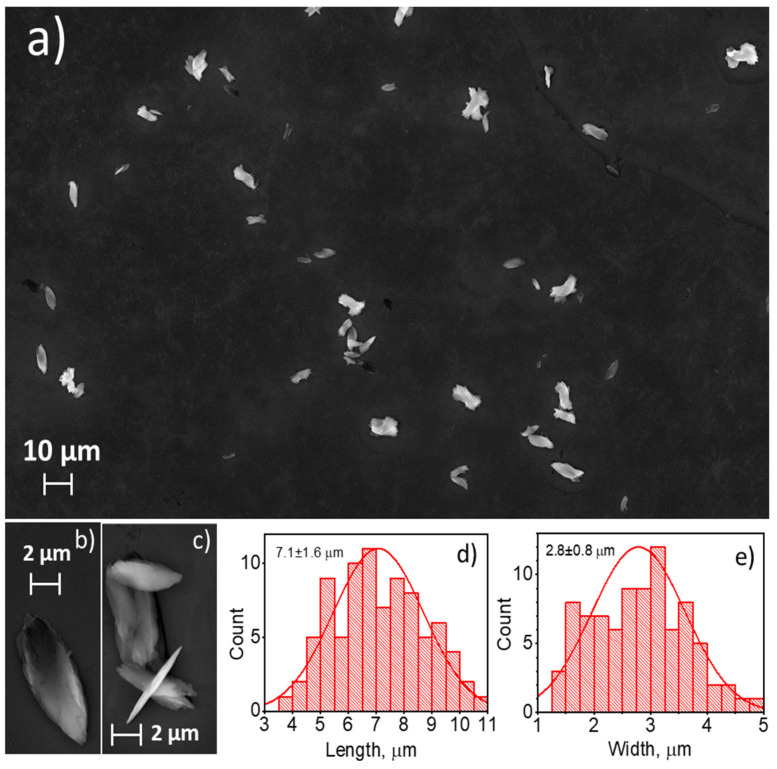
SEM images of Sample **2** (panels **a**–**c**). Particle size distribution (length and width) is shown in panels (**d**,**e**). The particles have the shape of the elliptic plates with ratio length:width:height of about 13:5:1. The average length and width were found to be equal to 7.1 ± 1.6 and 2.8 ± 0.8 µm, respectively.

**Figure 3:**
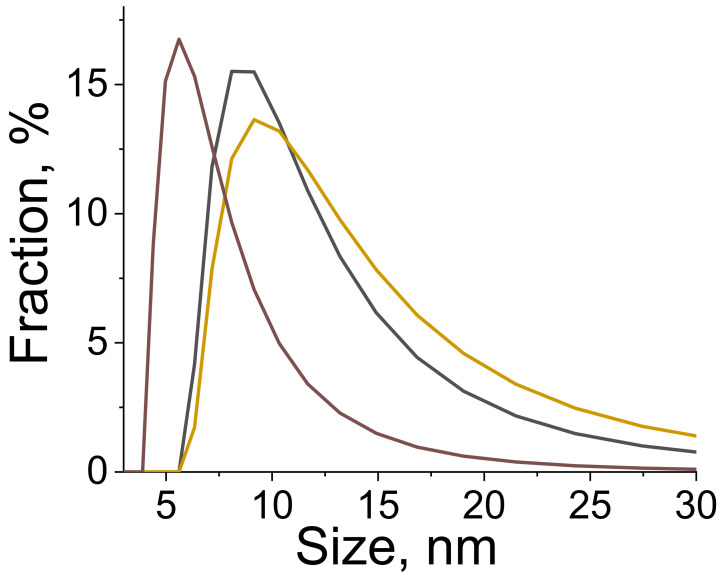
The particle size distribution of the aqueous solution of **3** is revealed by dynamic light scattering as a result of three parallel measurements. The average particle size is equal to about 8 ± 2 nm (spherical approximation).

A scanning electron microscope was used to observe the shape and the size of the particles in the synthesized materials. Sample **1**, which was synthesized by a slow mixing of equal volumes of sodium terephthalate (2 mM) and europium chloride (1 mM) aqueous solutions, precipitated in the form of a polycrystalline solid with the average particle size of 120 ± 30 µm (Figure 1). The observed species consisted of smaller particles stacked together forming dendrimer-like microparticle assemblies. The addition of the non-ionic surfactant (PEG-6000) to the reaction mixture and ultrasonication without a change in the Eu^3+^ and bdc^2−^ concentrations (Sample **2**) prevented the aggregation of the microparticles and resulted in the formation of individual microparticles (Figure 2a–c). The particles had a leaf-like shape with ratio length:width:height of about 13:5:1. The particles size was obtained from SEM images, the particle size distribution is shown in Figure 2d,e. The average length and width were calculated from these distributions and are equal to 7.1 ± 1.6 and 2.8 ± 0.8 µm, respectively. We found that under ultrasonication the solution remained clear to the eye when the concentration of Eu^3+^ and bdc^2−^ was decreased twofold (1 mM Na_2_bdc and 0.5 mM EuCl_3_) both in the absence and the presence of the surfactant (PEG-6000). We could not precipitate the solid from the reaction mixture using high-speed centrifugation or by salting-out using organic solvents. Therefore, the formation of the nano-sized particles of europium(III) terephthalate was supposed. In order to exclude the contribution of the PEG micelles to the experimental data, in further experiments we carefully studied the aqueous suspension of Sample **3** obtained by a slow mixing of equal volumes of the 1 mM Na_2_bdc and 0.5 mM EuCl_3_ accompanied by ultrasonication and vigorous stirring without a PEG-6000 addition. The particle size distribution was revealed by a dynamic light scattering technique, resulting in the average particle size equal to about 8 ± 2 nm (Figure 3). The SEM-EDX study of **3** aggregates formed by drying the reaction mixture on the silicon plate revealed the presence of Eu in the sample but did not determine the particle size due to the insufficient spatial resolution of the used SEM microscope. The direct observation of the species using TEM was also problematic because the high-energy electron radiation (>100 kV) burned out the sample due to the decomposition of an organic linker (terephthalate ion).

In our study, we have found that ultrasonication and PEG-6000 addition significantly decreases the particle size and prevents aggregation. The increase in particle size can be achieved via continuous growth of a particle or gradual aggregation of various particles or seed crystals. The contact of particles can reduce the total surface area in the aggregation process resulting in overall energy reduction. Ultrasonication can encourage surface tension between the species caused by the acoustic radiation force on a compressible particle [26]. The effect of PEG addition on the particle size can be explained by the well-known properties of surfactants including polyethylene glycol to be adsorbed on the particles or seed crystals that decrease their surface energy and prevents aggregation [27,28,29]. Surprisingly, we revealed that the twofold decrease in the reagents’ concentration leads to size reduction for several orders. A recent kinetic study of zinc-2-methylimidazole MOF ZIF-8 [30] reported that nucleation and crystal growth rates non-monotonously depend on the concentration of the reagents. During the low concentrations of the metal ions and the organic linker, the 1:1 M:L complex dominates. This state is called the “pre-equilibrium”. Further nucleation is associative and fast because the central atom has several weakly coordinated solvent molecules and can easily react with other 1:1 complexes resulting in the formation of oligomeric secondary building units (SBUs) [31]. Increasing the concentration of the metal ions and the organic linker leads to the domination of 1:2 and 1:3 M:L complexes. The aggregation of 1:2 and 1:3 M:L complexes into SBUs is slower than 1:1 complexes, which results in slower nucleation. Therefore, nucleation is faster than the growth process in solutions containing low concentrations of the metal ions and the organic linker, which explains the formation of the smaller particle size of the MOFs crystallizing at low concentrations.

### 3.2. Crystal Structure

**Figure 4:**
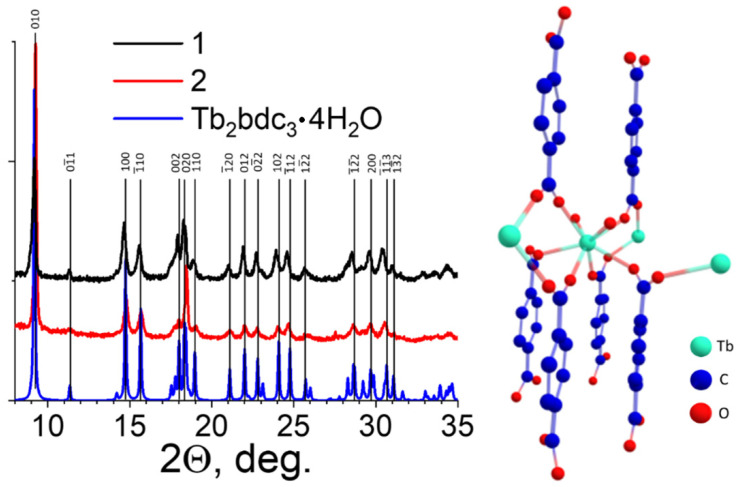
The XRD patterns of europium(III) terephthalate powders (**1** and **2**) and the simulated XRD pattern of Tb_2_bdc_3_·4H_2_O single-crystal structure taken from ref. [32] and the crystal structure of Tb_2_bdc_3_·4H_2_O.

The X-ray powder diffraction (XRD) patterns were measured (Figure 4) for Samples **1** and **2** to discover the crystalline phase of the obtained materials. We could not precipitate Sample **3** from the solution; therefore, the XRD pattern of Sample **3** was not measured. Analysis of XRD patterns demonstrated that synthesized materials **1** and **2** are isostructural with the Tb_2_bdc_3_·4H_2_O [32], the typical crystalline phase of lanthanide terephthalates [22], which indicated that materials **1** and **2** were obtained in a form of Eu_2_bdc_3_·4H_2_O. This structure is a three-dimensional metal-organic framework (MOF), where octacoordinated Eu^3+^-ions are bound to the two water molecules and six terephthalate ions through the oxygen atoms (Figure 4). XRD peaks of Eu_2_bdc_3_·4H_2_O in Samples **1** and **2** slightly diverge from their counterparts measured for Tb_2_bdc_3_·4H_2_O reported previously [32]. To compare the structures of Eu_2_bdc_3_·4H_2_O and Tb_2_bdc_3_·4H_2_O materials, the refinement of unit cell parameters was performed for the Eu_2_bdc_3_·4H_2_O samples (Table 2). One can observe that the structure of coarse-crystalline Eu_2_bdc_3_·4H_2_O (**1**) is slightly different from that of Tb_2_bdc_3_·4H_2_O. The ionic radius of the octacoordinated Eu^3+^ ion (1.066 Å) is slightly larger than that of the octacoordinated Tb^3+^ ion (1.040 Å) [33], which most likely results in minor differences between Eu_2_bdc_3_·4H_2_O and Tb_2_bdc_3_·4H_2_O structures. The unit cell parameters of microcrystalline Eu_2_bdc_3_·4H_2_O (**2**) are somewhat different, both from that of coarse-crystalline Eu_2_bdc_3_·4H_2_O (**1**) and Tb_2_bdc_3_·4H_2_O [32], which is likely caused by the surface defects due to the relatively small particle size of several micrometers. 

### 3.3. Luminescent Properties

**Figure 5:**
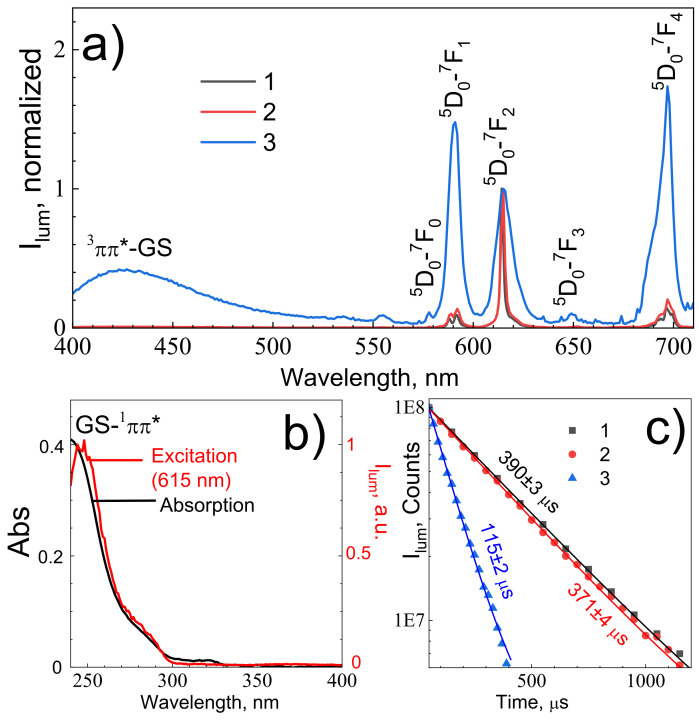
(**a**) The emission spectra of europium(III) terephthalate samples of different sized particles (λ_ex_ = 250 nm) normalized at the 615 nm emission band intensity. Sample numbers are shown in legend; (**b**) absorption (black line) and excitation (red line, λ_em_ = 615 nm) spectra of aqueous solution europium(III) terephthalate nanoparticles **3**; (**c**) 615 nm luminescence decay curves of europium(III) terephthalate samples of different sized particles.

Terephthalate ions are known to intensively absorb ultraviolet light, promoting them into the ^1^ππ* singlet electronic excited state [32,34,35]. In europium(III) terephthalate, the ^1^ππ* state efficiently undergoes the ^3^ππ* triplet electronic excited state by intersystem crossing due to the heavy atom effect [35] followed by an energy transfer to ^5^D_1_ level of the Eu^3+^ ion, due to relatively close energy values of the lowest energy ^3^ππ* excited state of terephthalate ion [35] (≈20,000 cm^−1^) and ^5^D_1_ level of Eu^3+^ ion [36] (≈19,000 cm^−1^). ^5^D_1_ level of the Eu^3+^ ion [36] then undergoes internal conversion followed by emission corresponding to ^5^D_0_–^7^F_J_ (J = 0–5) transitions. Figure 5a presents the emission spectra of the europium(III) terephthalate series (**1**–**3**) upon 250 nm excitation into the ^1^ππ* singlet electronic excited state of the terephthalate ion. The emission spectra include narrow lines corresponding to the transitions from excited ^5^D_0_ to lower ^7^F_J_ levels: ^5^D_0_–^7^F_0_ (578 nm),^5^D_0_-–^7^F_1_ (590 nm), ^5^D_0_–^7^F_2_ (615 nm), ^5^D_0_–^7^F_3_ (649 nm), and ^5^D_0_–^7^F_4_ (697 nm). The emission spectrum of nanocrystalline **3** also contains spectrally broad band peaking at about 420 nm, which corresponds to the terephthalate phosphorescence [35]. The most prominent transitions in the emission spectra are magnetic dipole ^5^D_0_–^7^F_1_ and forced electric dipole ^5^D_0_–^7^F_2_ and ^5^D_0_–^7^F_4_ transitions. The excitation spectrum (λ_em_ = 615 nm) of nanocrystalline **3** resembles its UV–Vis absorption spectrum (Figure 5b) consisting of a 250 nm band as well as a 280 nm shoulder corresponding to the transitions into ^1^ππ* singlet electronic excited states of the terephthalate ion. One can notice that emission bands corresponding to the f–f transitions of the Eu^3+^ ion significantly broaden with the particle size reduction. Thus, the ^5^D_0_–^7^F_2_ band of coarse-crystalline **1**, microcrystalline **2**, and nanocrystalline **3** have full width at half maximum (fwhm) equal to 48, 66, and 238 cm^−1^, respectively. The smaller particles have larger surface-to-volume ratio and the number of structural defects, which results in a larger dispersion of energies of electronic levels of Eu^3+^ ions caused by the larger non-uniformity of the local environment of europium ions [37,38]. The luminescence decay curves of europium(III) terephthalate (Figure 5c) are fitted by single-exponential functions:

(1) $$I_{lum}\left(t\right)=I_{o}e^{-\frac{t}{\tau_{f}}}$$

where time constant *τ_f_* corresponds to the observed lifetime of ^5^D_0_ level. The observed lifetime of ^5^D_0_ level of coarse-crystalline **1**, microcrystalline **2**, and nanocrystalline **3** were found to be equal to 393 ± 3, 371 ± 4, and 115 ± 2 μs, respectively.

Luminescence decay is affected by the combination of radiative and nonradiative processes. Radiative decay rate is determined by dipole transition strength and local-field correction. Nonradiative processes include multi-phonon relaxation, quenching on impurities (e.g., O-H group of water molecules) and cooperative processes (cross-relaxation, energy migration). Detailed descriptions of these processes were provided in our earlier papers [39,40]. The radiative and nonradiative decay rates of Eu^3+^-doped phosphors can be calculated from the emission spectrum using 4f–4f intensity theory [41]. Magnetic dipole ^5^D_0_–^7^F_1_ transition probability A_0-1_ = A_MD,0_∙n_0_^3^ = 14.65∙1.5^3^ = 49 s^−1^. A_MD,0_ is the spontaneous emission probability of the magnetic dipole ^5^D_0_–^7^F_1_, 14.65 s^−1^, and n_0_ is the refractive index, 1.5 [34]. Radiative decay rates A_0–λ_ (λ = 2, 4) of the ^5^D_0_–^7^F_λ_ emission transition can be obtained from this formula:

(2) $$A_{0-\lambda }=A_{0-1}\frac{\nu_{0-1}}{\nu_{0-\lambda }}\frac{I_{0-\lambda }}{I_{0-1}},\;$$

where I_0–λ_ and ν_0–λ_ are the integral intensity and frequency of the ^5^D_0_–^7^F_λ_ emission transition. The total radiative decay rate, A_r_, could be calculated by summing all the A_0–λ_ radiative decay rates (λ = 1, 2, 4). The total decay rate is reciprocal to the observed lifetime of ^5^D_0_ level, shown in Figure 5c, $A_{total}=\frac{1}{\tau_{f}}$, whereas the nonradiative probability can be calculated as: $A_{nr}=A_{total}-A_{r}$. Quantum efficiency of ^5^D_0_ level is $\eta =\frac{A_{r}}{A_{total}}$. Decay rates and quantum efficiencies of the ^5^D_0_ level of europium(III) terephthalates **1**–**3** are summarized in Table 3.

Analyzing Table 2, one can see the quantum efficiencies of the ^5^D_0_ level and Eu^3+^ luminescence quantum yields decrease in series **1**–**3** simultaneously with particle size, whereas nonradiative decay rate constants increase upon the size reduction. Smaller particles have larger surface-to-volume ratios, resulting in more efficient quenching of the ^5^D_0_ level and Eu^3+^ by the water molecules in an aqueous solution [42]. Comparing the quantum efficiencies of the ^5^D_0_ level and Eu^3+^ luminescence quantum yields values, one can notice that the η/Φ ratio is equal to 0.5–0.9, which indicates a very efficient energy transfer from initially excited terephthalate chromophore to the ^5^D_0_ level of Eu^3+^ ion.

### 3.4. Sensing Transition Metal Cations

**Figure 6:**
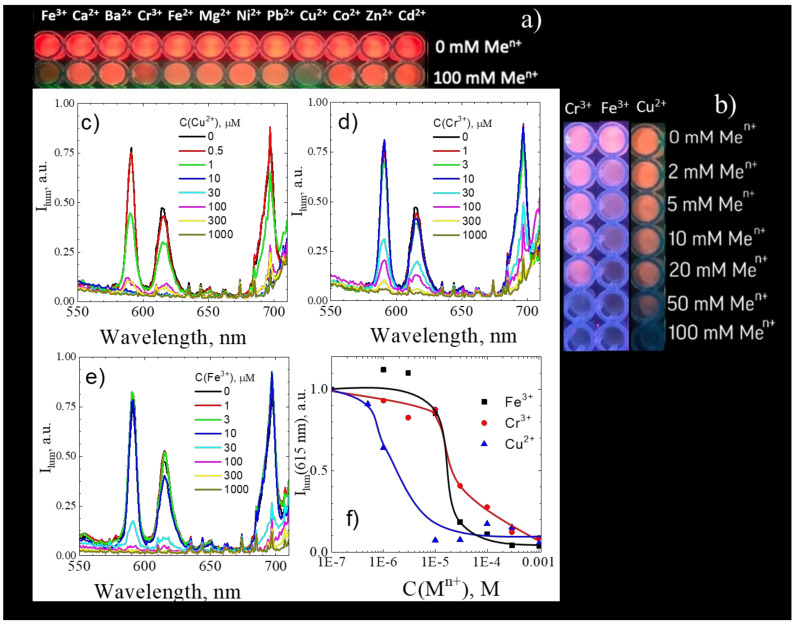
(**a**,**b**) Photographs of aqueous suspension of coarse-crystalline **1** under 254 nm illumination in the absence and presence of various metal ions; emission spectra of aqueous solution of nanocrystalline **3** in the absence and presence of various concentrations of Cu^2+^ (**c**), Cr^3+^ (**d**), and Fe^3+^ (**e**) ions upon 250 nm excitation; (**f**) Cu^2+^, Cr^3+^, and Fe^3+^ concentration dependence of 615 nm emission intensity of Sample **3**.

Previous studies demonstrated that the presence of impurities such as ions of transition metals (Fe^3+^, Cu^2+^, Pb^2+^, MnO_4_^−^, Cr_2_O_7_^2−^) [43], and organic compounds (aromatic, nitroaromatic, carbonyl compounds) can significantly quench the luminescence of the Eu-based metal-organic frameworks [1,2,3,4,5,6,7,8,9] making them prospective for the design of luminescent sensors for various pollutants and explosives. To reveal the selectivity of the europium(III) terephthalate MOF luminescence quenching to the various metal cations, 80 μL of aqueous suspensions of coarse-crystalline **1** (C(Eu^3+^) = 8 mM) was mixed with the 100 μL of metal salt solutions (C(M^n+^) = 100 mM; M^n+^ = Fe^3+^, Ca^2+^, Ba^2+^, Cr^3+^, Fe^2+^, Mg^2+^, Ni^2+^, Pb^2+^, Cu^2+^, Co^2+^, Zn^2+^, Cd^2+^) or distilled water. After 30 min, the photographs of these solutions under 254 nm illumination were recorded (Figure 6a,b). It was found that the Eu-based red emission faded only in the presence of Fe^3+^, Cr^3+^ and Cu^2+^ ions (Figure 6a) starting from metal ion concentration 10–50 mM (Figure 6b). The emission spectra of aqueous solutions of nanocrystalline **3** (C(Eu^3+^) = 5 μM) in the absence and in the presence of various concentrations of Cu^2+^, Cr^3+^, and Fe^3+^ ions (λ_exc_ = 250 nm) indicate the quenching of Eu^3+ 5^D_0_–^7^F_λ_ luminescence by the above-mentioned metal ions (Figure 6c–e). The dependence of the 615 nm emission band intensity on the Cu^2+^, Cr^3+^, and Fe^3+^ concentration is given in Figure 6f. The concentration dependence resembles the step-function, where luminescence intensity sharply falls starting from the certain concentration of metal ion: 1 μM of Cu^2+^ and 30 μM of Cr^3+^ or Fe^3+^. Surprisingly, we revealed that the addition of Fe^3+^ ions resulted in simultaneous quenching for the Eu^3+ 5^D_0_–^7^F_λ_ luminescence (591, 615, and 697 nm bands) and the terephthalate phosphorescence (420 nm), whereas the addition of Cu^2+^ and Cr^3+^ ions almost failed to reduce the intensity of the terephthalate phosphorescence band at 420 nm (Figures S1–S3, Supplementary Materials). This observation indicates a different quenching mechanism of Eu^3+ 5^D_0_–^7^F_λ_ luminescence by the above-mentioned metal ions. Most likely, Cu^2+^, Cr^3+^, and Fe^3+^ ions somehow coordinate with the oxygens of terephthalate ligands, but Fe^3+^ ions quench the ^3^ππ* triplet electronic excited state of terephthalate ion, whereas Cu^2+^ and Cr^3+^ ions quench the ^5^D_0_ level of Eu^3+^. To reveal the complete quenching mechanism, one must study the excited-state dynamics of singlet and triplet electronic states of terephthalate ion, as well as the ^5^D_0_ level of Eu^3+^, depending on the heavy metal ion concentration by time-resolved transient absorption and luminescence spectroscopy methods. We have found that nanocrystalline europium(III) terephthalate MOF **3** demonstrates significantly lower limits of detection on Cu^2+^, Cr^3+^, and Fe^3+^ ions than coarse-crystalline **1** (10–50 mM for coarse-crystalline **1** vs 1–30 μM for nanocrystalline **3,** Figure 6b,f). This observation is explained by a larger surface-to-volume ratio of nanoparticles relatively to the bulk material, resulting in a higher luminescence quenching efficiency of the later materials due to a greater number of coordination sites. The sensitivity of our materials to Cu^2+^, Cr ^3+^ and Fe^3+^ ions is comparable with the best reported luminescent MOF-based sensors reported previously (Table 4). Despite the higher sensitivity of electrochemical MOF-based sensors (Table 4), the luminescent sensors can be used for the design of relatively inexpensive express tests on heavy metal ions.

## 4. Conclusions

In summary, we reported the ultrasound-assisted wet-chemical synthesis and characterization of luminescent coarse-, micro-, and nano-crystalline Eu_2_bdc_3_·4H_2_O MOFs. The particles of coarse-crystalline Eu_2_bdc_3_·4H_2_O, which are synthesized by the mixing of sodium terephthalate and europium chloride aqueous solutions without ultrasound, are dendrimer-like microparticle assemblies with the average particle size of 120 ± 30 µm. The microcrystalline MOFs were prepared by mixing sodium terephthalate and europium chloride aqueous solutions with the addition of PEG-6000 in the presence of ultrasonication. The microparticles have the shape of leaf-like plates and an average size of 7.1 × 2.8 µm. The average size of Eu_2_bdc_3_·4H_2_O nanoparticles, synthesized by the mixing of low-concentration sodium terephthalate and europium chloride aqueous solutions in the presence of ultrasonication, is equal to about 8 ± 2 nm. Thus, the reported Eu_2_bdc_3_·4H_2_O nanoparticles are the smallest nanosized rare-earth-based MOF crystals, to the best of our knowledge. The emission spectra of synthesized materials exhibit narrow lines corresponding to transitions from excited ^5^D_0_ to lower ^7^F_J_ levels of Eu^3+^ ion: ^5^D_0_–^7^F_0_ (578 nm),^5^D_0_–^7^F_1_ (590 nm), ^5^D_0_–^7^F_2_ (615 nm), ^5^D_0_–^7^F_3_ (649 nm), and ^5^D_0_–^7^F_4_ (697 nm). Size reduction resulted in a broadening of the emission bands. The Eu^3+^ luminescence quantum yields, upon excitation into ^1^ππ* singlet electronic excited state of terephthalate ion, were found to be of 10 ± 1%, 5 ± 1% and 1.5 ± 0.5% for coarse-, micro- and nanocrystalline Eu_2_bdc_3_·4H_2_O MOFs, respectively. The nonradiative decay rate of nanocrystalline europium(III) terephthalate was significantly larger that the corresponding values of Eu_2_bdc_3_·4H_2_O MOFs, which resulted from more efficient quenching of the ^5^D_0_ level and Eu^3+^ by the water molecules in aqueous solution due to greater surface-to-volume ratio of nanocrystalline MOF. The Cu^2+^, Cr^3+^, and Fe^3+^ ions efficiently and selectively quench the Eu^3+ 5^D_0_−^7^F_λ_ luminescence of nanocrystalline Eu_2_bdc_3_·4H_2_O MOFs starting from the relatively low concentrations of metal ion: 1 μM of Cu^2+^ and 30 μM of Cr^3+^ or Fe^3+^. The reported nanocrystalline europium(III) terephthalateis one of the most sensitive luminescent MOF-based sensorfor Cu^2+^, Cr ^3+^ and Fe^3+^ ions (Table 4). Therefore, synthesized nanocrystalline Eu_2_bdc_3_·4H_2_O MOFs can be considered promising luminescent probes for heavy metal ions in waste and drinking water.

## Figures and Tables

**Table 1:** Overview of the synthesis of europium(III) terephthalates **1**–**3**.

Sample	C(EuCl_3_)	C(Na_2_bdc)	PEG-6000	Ultrasonication	Stirring
**1**	1 mM	2 mM	-	-	+
**2**	1 mM	2 mM	20%	+	+
**3**	0.5 mM	1 mM	-	+	+

**Table 2:** Unit cell parameters for Tb_2_bdc_3_·4H_2_O [32] and Eu_2_bdc_3_·4H_2_O (Samples **1** and **2**).

Sample	a, Å	b, Å	c, Å	α, deg.	β, deg.	γ, deg.	V, Å^3^
Tb_2_bdc_3_·4H_2_O	6.14	10.07	10.10	102.25	91.12	101.52	596.63
Eu_2_bdc_3_·4H_2_O (**1**)	6.20	9.85	10.29	102.15	89.75	105.10	592.91
Eu_2_bdc_3_·4H_2_O (**2**)	6.16	9.8	10.22	101.84	90.27	104.86	582.19

**Table 3:** Radiative (**A_r_**), nonradiative (**A_nr_**) and total (**A_total_**) decay rates, quantum efficiencies (**η**) of the ^5^D_0_ level of europium(III) and Eu^3+^ luminescence quantum yields (**Φ)** upon excitation into ^1^ππ* singlet electronic excited state of terephthalate ion terephthalates **1**–**3**.

Sample	A_r_ (s^−1^)	A_nr_ (s^−1^)	A_total_ (s^−1^)	η (%)	Φ (%)
**1**	371	2193	2564	14.5	10 ± 1
**2**	290	2405	2695	10.8	5 ± 1
**3**	150	8545	8695	1.7	1.5 ± 0.5

**Table 4:** Limits of detection (LOD) of nanocrystalline europium(III) terephthalate tetrahydrate **3** and previously reported materials for Cu^2+^, Cr ^3+^ and Fe^3+^ ions.

Sensing Material	Method	Target Contaminant	LOD	Ref.
Eu_2_(bdc)_3_·4H_2_O	luminescent	Cu^2+^	1 μM	Current work
Tb(BTC)(H_2_O)	luminescent	Cu^2+^	10 μM	[2]
CDs@Eu-DPA MOFs	luminescent	Cu^2+^	26.3 nM	[3,43]
[Eu(PDC)_1.5_(DMF)]·(DMF)_0.5_(H_2_O)_0.5_	luminescent	Cu^2+^	10 mM	[7,44]
Eu_2_(FMA)_2_(OX)(H_2_O)_4_·4H_2_O	luminescent	Cu^2+^	100 μM	[7,45]
[Eu_4_(BPT)_4_(DMF)_2_(H_2_O)_8_]	luminescent	Cu^2+^	10 μM	[7,46]
[Tb_3_(L)_2_(HCOO)(H_2_O)_5_]·DMF·4H_2_O	luminescent	Cu^2+^	100 μM	[7,47]
[Eu(ox)_2_(H_2_O)](Me_2_NH_2_)(H_2_O)_3_	luminescent	Cu^2+^	10 μM	[10,48]
Zr_6_(O)_8_(OH_2_)_8_(tpdc)_4_	luminescent	Cu^2+^	1 μM	[10,49]
PCN-222-Pd(II)	luminescent	Cu^2+^	50 nM	[10,50]
Me_2_NH_2_@MOF-1	electrochemical	Cu^2+^	10 pM	[3,51]
Eu_2_(bdc)_3_ nanoparticles	luminescent	Fe^3+^	30 μM	Current work
[Me_2_NH_2_][In(abtc)]·solvents	luminescent	Fe^3+^	34.5 μM	[52]
[LnK(BPDSDC)(DMF)(H_2_O)]·x(solvent)	luminescent	Fe^3+^	10 μM	[10,53]
[Eu(BTPCA)(H_2_O)]·2DMF·3H_2_O	luminescent	Fe^3+^	10 μM	[7,54]
[Eu(HL)(H_2_O)_2_]_n_·2H_2_O	luminescent	Fe^3+^	1 μM	[7,55]
EuL	luminescent	Fe^3+^	100 μM	[7,56]
[H_2_NMe_2_]_3_[Tb(DPA)_3_]	luminescent	Fe^3+^	10 μM	[7,57]
Eu (4′-(4-carboxyphenyl)-2,2′: 6′,2″-terpyridine)_3_	luminescent	Fe^3+^	100 μM	[7,58]
[H(H_2_O)_8_][DyZn_4_(imdc)_4_(im)_4_]	luminescent	Fe^3+^	1 mM	[7,59]
Eu^3+^@Ga_2_(OH)_4_(C_9_O_6_H_4_)	luminescent	Fe^3+^	0.28 μM	[7,60]
nTbL	luminescent	Fe^3+^	10 μM	[7,61]
[Eu(atpt)_1.5_(phen)(H_2_O)]_n_	luminescent	Fe^3+^	500 μM	[7,62]
[(CH3)_2_NH_2_] ·[Tb(bptc)]·xS	luminescent	Fe^3+^	10 μM	[7,63]
Tb-BTB	luminescent	Fe^3+^	10 μM	[7,64]
[Eu_3_(BDC)_4.5_(H_2_O)(DMF)_2_]	luminescent	Fe^3+^	1 μM	[7,65]
[Cd(L)(BPDC)]·2H_2_O	luminescent	Fe^3+^	2 μM	[8,66]
[Cd(L)(SDBA)(H_2_O)]∙0.5H_2_O	luminescent	Fe^3+^	2 μM	[8,66]
[Zn5(hfipbb)_4_(trz)_2_(H_2_O)_2_]	luminescent	Fe^3+^	10 μM	[8,67]
[Eu(Hpzbc)_2_(NO_3_)]·H_2_O	luminescent	Fe^3+^	10 μM	[8,68]
[Eu(L)(H_2_O)_2_]·NMP·H_2_O	luminescent	Fe^3+^	100 nM	[8,69]
[Tb(L1)_1.5_(H_2_O)]·3H_2_O	luminescent	Fe^3+^	10 μM	[10,70]
Bisdiene macrocycle	luminescent	Fe^3+^	0.58 μM	[71]
2-(cyclohexylamino)-3-phenyl-4Hfuro [3,2-c]chromen-4-one	luminescent	Fe^3+^	1.73 μM	[72]
[Me_2_NH_2_][In(abtc)]·solvents	luminescent	Fe^3+^	34.5 μM	[53]
PPCOT/NiFe_2_O_4_/C-SWCNT	electrochemical	Fe^3+^	100 pM	[73]
Eu_2_(bdc)_3_ nanoparticles	luminescent	Cr^3+^	30 μM	Current work
Tb(BTC)(H_2_O)	luminescent	Cr^3+^	10 μM	[2]
[TbK(BPDSDC)(DMF)(H_2_O)_2_]			10 μM	[8,74]
[Eu_2_L_3_(DMF)_3_]·2DMF·5H_2_O	luminescent	Cr^3+^	75.2 nM	[75]
ATNA deriviative	electrochemical	Cr^3+^	130 pM	[76]

## Data Availability

Data is contained within this article and corresponding supplementary materials.

## Supplementary Materials

The following are available online at https://www.mdpi.com/article/10.3390/nano11092448/s1, Figure S1: (a) Emission spectra of aqueous solution of nanocrystalline **3** in the absence and presence of various concentrations of Cu^2+^ upon 250 nm excitation; (b) Cu^2+^ concentration dependence of 420, 591, 615, and 697 nm emission intensities of **3**, Figure S2: (a) Emission spectra of aqueous solution of nanocrystalline **3** in the absence and presence of various concentrations of Cr^3+^ upon 250 nm excitation; (b) Cr^3+^ concentration dependence of 420, 591, 615, and 697 nm emission intensities of **3**, Figure S3: (a) Emission spectra of aqueous solution of nanocrystalline **3** in the absence and presence of various concentrations of Fe^3+^ upon 250 nm excitation; (b) Fe^3+^ concentration dependence of 420, 591, 615, and 697 nm emission intensities of **3**.
