# Supplementary material for: Ultrasound-Assisted Synthesis of Luminescent Micro- and Nanocrystalline Eu-Based MOFs as Luminescent Probes for Heavy Metal Ions
Source: Nanomaterials (Basel). 2021 Sep 20;11(9):2448. doi: 10.3390/nano11092448 (PMC8468986; doi:10.3390/nano11092448)
Supplement: Supplementary file 1 [file nanomaterials-11-02448-s001.zip › nanomaterials-1335058-supplementary.pdf]

nanomaterials

Supplementary Materials

Ultrasound-Assisted Synthesis of Luminescent Micro- and Nanocrystalline Eu-based MOFs as Luminescent Probes for Heavy Metal Ions

Stefaniia S. Kolesnik[1], Viktor G. Nosov[1], Ilya E. Kolesnikov[1], Evgenia M. Khairullina[1], Ilya I. Tumkin[1], Aleksandra A. Vidyakina[1], Alevtina A. Sysoeva[2], Mikhail N. Ryazantsev[1,3], Maxim S. Panov[1], Vasiliy D. Khripun[1], Nikita A. Bogachev[1], Mikhail Yu. Skripkin[1], Andrey S. Mereshchenko[1,2,*]

[1] Saint-Petersburg State University, 7/9 Universitetskaya emb., St. Petersburg 199034, Russia; staphylinuscaesareus@gmail.com (S.S.K.); nosoff.vitia2018@yandex.ru (V.G.N.); ilya.kolesnikov@spbu.ru (I.E.K); iskint@mail.ru (E.M.K.); i.i.tumkin@spbu.ru (I.I.T.); vidyakina.aleksandra@mail.ru (A.A.V); mikhail.n.ryazantsev@gmail.com (M.N.R.); m.s.panov@spbu.ru (M.S.P.); v.khripun@spbu.ru (V.D.K.); allanfrack@yandex.ru (N.A.B); skripkin1965@yandex.ru (M.Yu.S.); a.mereshchenko@spbu.ru (A.S.M)

[2] Sirius University of Science and Technology, 1 Olympic Ave, 354340, Sochi, Russia; sysoevaaa_04@mail.ru (A.A.S)

[3] Saint Petersburg Academic University, ul. Khlopina 8/3, 194021, St. Petersburg, Russia

* Correspondence: a.mereshchenko@spbu.ru; Tel.: +7-951-677-5465

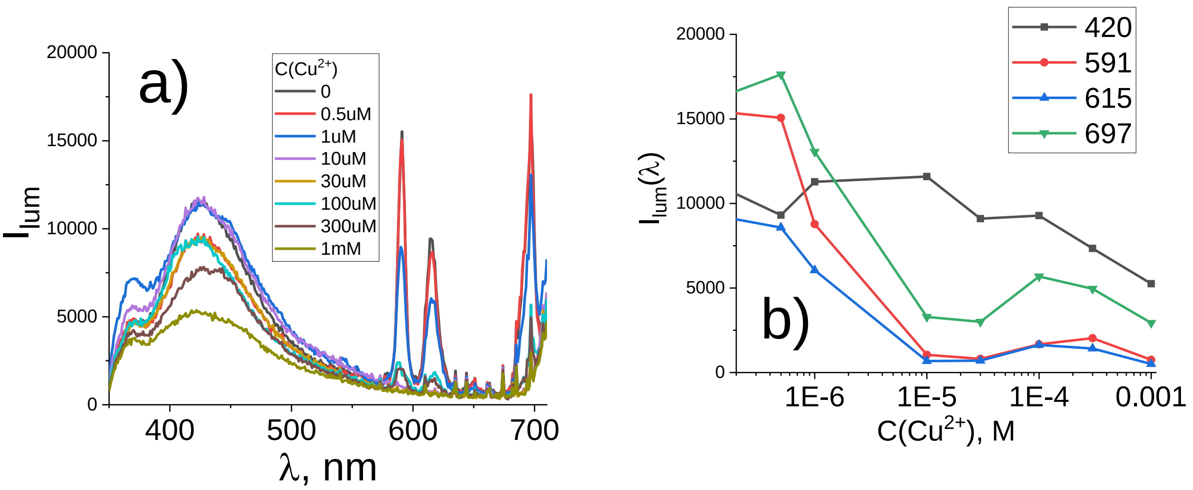

Figure S1. **(a)** Emission spectra of aqueous solution of nanocrystalline **3** in the absence and presence of various concentrations of Cu^{2+} upon 250-nm excitation; (b) Cu^{2+} concentration dependence of 420, 591, 615, and 697-nm emission intensities of **3.**

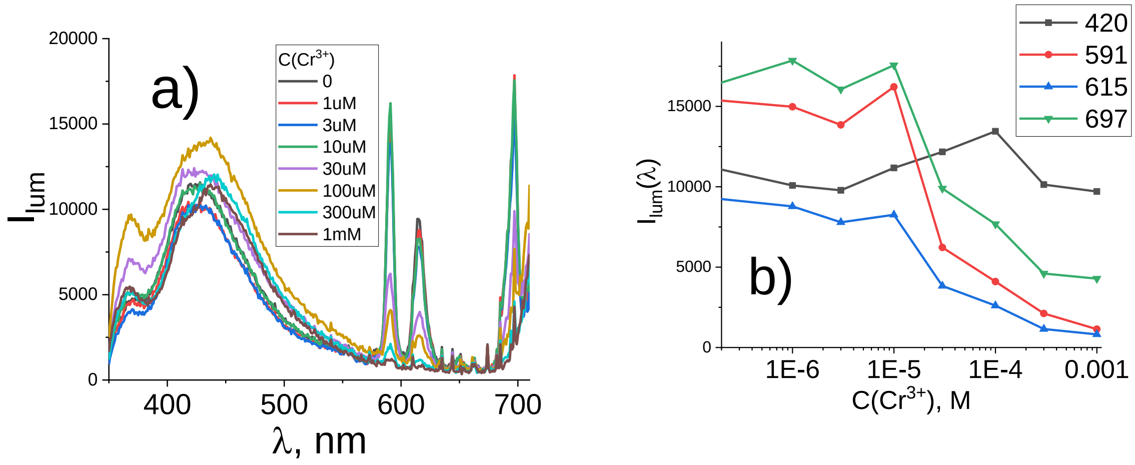

Figure S2. (a) Emission spectra of aqueous solution of nanocrystalline **3** in the absence and presence of various concentrations of Cr^{3+} upon 250-nm excitation; (b) Cr^{3+} concentration dependence of 420, 591, 615, and 697-nm emission intensities of **3.**

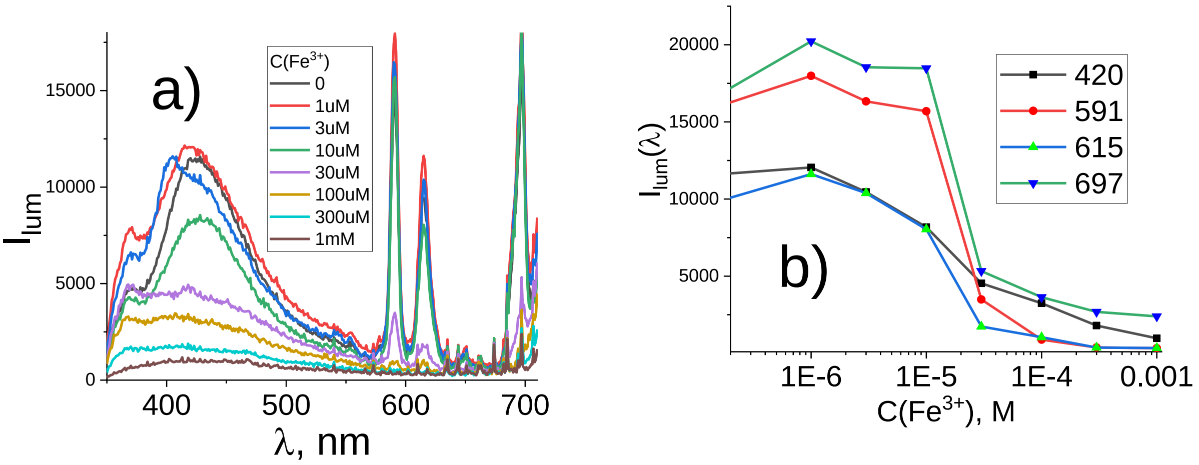

Figure S3. (a) Emission spectra of aqueous solution of nanocrystalline **3** in the absence and presence of various concentrations of Fe^{3+} upon 250-nm excitation; (b) Fe^{3+} concentration dependence of 420, 591, 615, and 697-nm emission intensities of **3.**
